# Gut microbiota facilitates adaptation of the plateau zokor (*Myospalax baileyi*) to the plateau living environment

**DOI:** 10.3389/fmicb.2023.1136845

**Published:** 2023-02-24

**Authors:** Bin Hu, Jiamin Wang, Ying Li, Jin Ge, Jinchao Pan, Gaojian Li, Yongcai He, Haishun Zhong, Bo Wang, Yanyi Huang, Shuyi Han, Yanan Xing, Hongxuan He

**Affiliations:** ^1^Institute of Zoology, Chinese Academy of Sciences, Beijing, China; ^2^College of Life Science, University of Chinese Academy of Sciences, Beijing, China; ^3^College of Agriculture and Animal Husbandry, Qinghai University, Xining, China; ^4^College of Animal Sciences, Anhui University of Science and Technology, Huainan, China; ^5^Animal Husbandry and Veterinary Station of Xunhua, Xining, Qinghai, China

**Keywords:** plateau zokor (*Myospalax baileyi*), gut microbiota, metagenome, plateau adaptation, SCFAs, TPPII

## Abstract

Gut microbiota not only helps the hosts to perform many key physiological functions such as food digestion, energy harvesting and immune regulation, but also influences host ecology and facilitates adaptation of the host to extreme environments. Plateau zokors epitomize successful physiological adaptation to their living environment in the face of the harsh environment characterized by low temperature, low pressure and hypoxia in the Tibetan plateau region and high concentrations of CO_2_ in their burrows. Therefore, here we used a metagenomic sequencing approach to explore how gut microbiota contributed to the adaptive evolution of the plateau zokor on the Qinghai-Tibet Plateau. Our metagenomic results show that the gut microbiota of plateau zokors on the Tibetan plateau is not only enriched in a large number of species related to energy metabolism and production of short-chain fatty acids (SCFAs), but also significantly enriched the KO terms that involve carbohydrate uptake pathways, which well address energy uptake in plateau zokors while also reducing inflammatory responses due to low pressure, hypoxia and high CO_2_ concentrations. There was also a significant enrichment of tripeptidyl-peptidase II (TPPII) associated with antigen processing, apoptosis, DNA damage repair and cell division, which may facilitate the immune response and tissue damage repair in plateau zokors under extreme conditions. These results suggest that these gut microbiota and their metabolites together contribute to the physiological adaptation of plateau zokors, providing new insights into the contribution of the microbiome to the evolution of mammalian adaptation.

## 1. Introduction

Gut microbiota can help the host perform many key physiological functions such as food digestion, energy harvesting and immune regulation (Tsuchida et al., 2004; Tremaroli and Bäckhed, 2012; Amato et al., 2014; Zhang et al., 2015). It may also strongly affect host ecology, for example, commensal microbes in animal’s guts often help to exclude bacterial pathogens (Łukasik et al., 2013; Steele et al., 2021), and increase their tolerance to extreme environments (Zhang et al., 2018). For example, one study showed that the rumen microbiome produced more methane and volatile fatty acids to help Tibetan sheep (*Ovis aries*) and yak (*Bos grunniens*) adapt to the harsh high-altitude environment (Zhang et al., 2016). The Qinghai-Tibet Plateau is the highest plateau in the world, known as the “roof of the world” and the “third pole” (Xu et al., 2011). Environmental factors such as temperature and topography in the Plateau have had great influences on the structure and evolution of animal populations in the region, leading to the evolution of unique species or specific subgroups with different physiological and genetic adaptations to lower altitude species (Yang et al., 2009; Zeng et al., 2020; Zhang G. et al., 2021). Although some native mammals on the Tibetan Plateau may be well adapted to extreme climates how the structure and function of animal gut microbial communities contribute to adaptation of the host to extreme environments is not fully understood.

The plateau zokor (*Myospalax baileyi*) is a small underground rodent endemic to China, mainly inhabiting alpine meadows, alpine grasslands, scrub and farmland on the Tibetan Plateau and surrounding high altitude areas, living alone in sealed underground burrows at altitudes of 2,000–4,200 m (Su et al., 2015; Zhang et al., 2022). Due to the confines of underground burrows and the effects of seasonal plant dieback, plateau zokor feed primarily on the underground parts of roots, rhizomes and other weeds, even preferring some common poisonous weeds such as *Oxytropis kansuensis* and *Stellera chamaejasme* (Liu D. et al., 2021; Kang et al., 2022). They are the epitome of successful physiological adaptation in the face of the harsh environment characterized by low temperatures, low pressure, lack of oxygen at high-altitude and high concentrations of CO_2_ in caves, as well as complete darkness (Pu et al., 2019a; Kang et al., 2020). The adaptations of plateau zokors to low oxygen conditions in the Tibetan plateau have been partially studied (Xu et al., 2021; Zhang T. et al., 2021).

Grassland small mammals often span large geographical areas and utilize different food resources, so host gut microbiota may show marked differences between habitats. Many animals have microbial communities in their habitats that play a key role in host biology, which influence many aspects of host health and have the potential to adapt to harsh environmental (West et al., 2019; Liu et al., 2021). While 16S ribosomal RNA (16S rRNA) sequencing allowed reliable taxonomic resolution down to the species level, it did not provide information on functional characteristics. Metagenomic research goes far beyond traditional 16S rRNA microbiome sequencing, enabling not only more accurate species classification, but also in-depth bioinformatics analysis (Mack et al., 2020), which provides a unique opportunity to explore the composition and function of the gut microbiota in response to environmental adaptation of the host.

In this study, we selected plateau zokors (*Myospalax baileyi*) and plateau pikas (*Ochotona curzoniae*) from alpine meadow ecosystems on the Tibetan Plateau and Brandt’s vole (*Lasiopodomys brandtii*), Mongolian gerbil (*Meriones unguiculatus*) and Daurian ground squirrel (*Spermophilus dauricus*) from typical grassland ecosystems in the Inner Mongolian grasslands as our subjects. In evolutionary terms, unlike the other four host animal species, which all belong to the *Rodentia*, the plateau pika belongs to the *Ochotonidae* of the *Lagomorpha*. The plateau zokor belongs to the *Spalacidae* of the *Rodentia*, the Brandt’s vole and the Mongolian gerbil belong to the *Circetidae* of the *Rodentia*, and the Daurian ground squirrel belongs to the *Sciuridae* of the *Rodentia*. These species represent the best adapted ecological populations of small mammals inhabiting grassland ecosystems at different altitudes. The gut contents of these target animal species were then analyzed by metagenome sequencing to assess the characteristics of the gut microbiota and to explore the relationship between adaptations of the host to its living environment and the gut microbiota.

## 2. Materials and methods

### 2.1. Ethics statement

This study was conducted in accordance with the Guidelines for the Care and Use of Animals in Research published by the Institute of Zoology, Chinese Academy of Sciences. This study was reviewed and approved by the Animal Ethics Committee of the Institute of Zoology, Chinese Academy of Sciences (2019FY100300-03).

### 2.2. Sample selection and sampling location information

**Figure 1:**
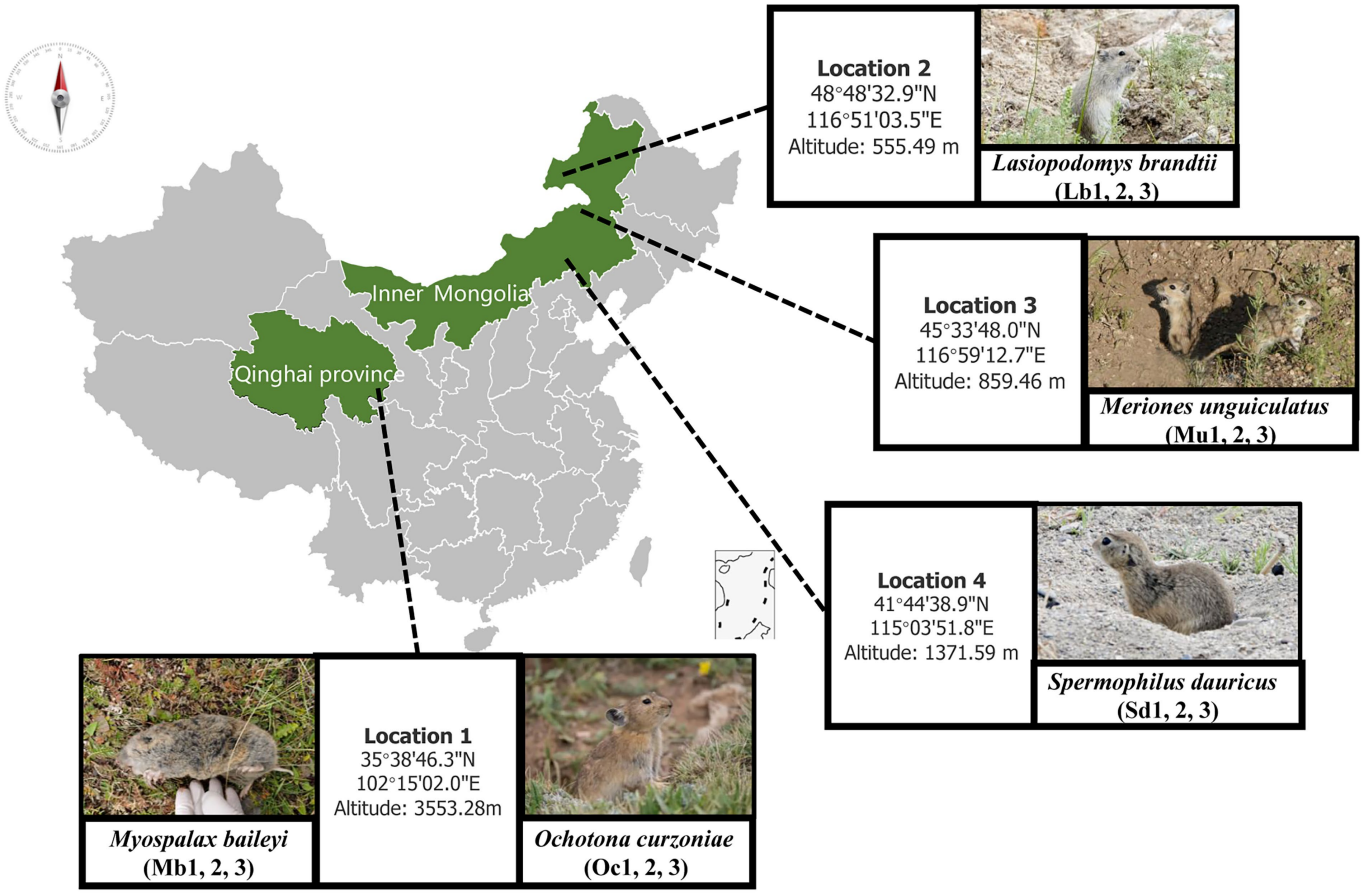
Sampling sites and host animal species names.

The samples were collected between 15 July and 15 August 2021. Wild plateau zokor (Mb) and plateau pika (Oc) were captured from Xunhua County (35°38′46.3″N 102°15′02.0″E), Qinghai Province, on the Qinghai-Tibet Plateau. During the same time period that we sampled the Tibetan Plateau, we collected the best adapted ecological populations of small mammals at three different lower elevations in the grasslands of Inner Mongolia. We captured samples of Brandt’s vole (Lb) at New Barag Right Banner (45°33′48.0″N 116°59′12.7″E), Mongolian gerbil (Mu) at East Ujimqin Banner (48°48′32.9″N 116°51′03.5″E), and then zaiTaibus Banner for Daur’s gopher (Sd) (41°44′38.9″N 115°03′51.8″E), respectively. The species and location information are shown in Figure 1; Table 1. Rope trapping method was used to trap plateau zokors, and then four other species were sampled using cage trapping. Captured Animal samples were euthanized with isoflurane and then taken back to the local laboratory for risk assessment. After confirming that there was no potential biosafety risk, the autopsy was performed and recorded information such as location, age, sex, and weight. The cecal contents were collected into 2 ml sterilized storage tubes, immediately stored in liquid nitrogen, immersed in dry ice during transport, and then stored in a laboratory freezer at –80°C. Then, cecum feces of three adult male individuals were selected for subsequent metagenomic sequencing analysis.

**Table 1:** Sampling location and sample data information.

Sampling location	Longitude and latitude	Altitude (meter)	Species	Gender
1 Xunhua Salar Autonomous County	35°38′46.3′′N 102°15′02.0″E	3553.28 m	*Myospalax baileyi*/*Ochotona curzoniae*	Male
2 New Barag Right Banner	45°33′48.0′′N 116°59′12.7″E	555.49 m	*Lasiopodomys brandtii*	Male
3 East Ujimqin Banner	48°48′32.9′′N 116°51′03.5″E	859.46 m	*Meriones unguiculatus*	Male
4 Taibus Banner	41°44′38.9′′N 115°03′51.8″E	1371.59 m	*Spermophilus dauricus*	Male

### 2.3. Extraction of genomic DNA

Total fecal genomic DNA was extracted from the cecum feces using the Fecal Genomic DNA Extraction Kit (TianGen) following the manufacturer’s protocol. DNA was analyzed for purity and integrity using agarose gel electrophoresis (AGE); DNA concentration was accurately quantified by Qubit 2.0. Then it was sent to Tianjin Nuohezhiyuan Bio-Information Technology Co., Ltd. for purification and sequencing.

### 2.4. Library construction and on-machine sequencing

The total genomic DNA samples were randomly broken into fragments of approximately 350 bp in length using a Covaris ultrasonic breaker, and the libraries were prepared by end-repair, A-tailing, sequencing junction, purification and Polymerase Chain Reaction (PCR) amplification. After library construction, the library was initially quantified using Qubit 2.0 and diluted to 2 ng/μL, and then the insert size of the library was checked using Agilent 2,100. After the insert size meets expectations, use the Q-PCR method to determine the effective concentration of the library. Accurate quantification (library effective concentration > 3 nM) to ensure library quality. After ensuring that the quality of the library is qualified, the different libraries are pooled according to the requirements of effective concentration and target data volume, and then Illumina PE150 sequencing is performed.

### 2.5. Data quality control

In order to ensure the accuracy and reliability of the results of the subsequent information analysis, the raw data should first be filtered by quality control and hosts to obtain Clean Data. Remove reads containing a certain percentage of low-quality bases (quality <=38) or more (default is set to 40 bp). Remove reads with Adapter removes reads containing more than a certain percentage (default 40 bp) of low-quality bases (mass < =38). Remove the reads whose N base reaches a certain proportion (the default is 10 bp). Remove the reads whose overlap with the Adapter exceeds a certain threshold (the default is 15 bp).

### 2.6. Gene assembly and prediction

Metagenome assembly was performed from the Clean Data of each sample after quality control. ORF (Open Reading Frame) was performed from the scaftigs (≥500 bp) of single sample assembly using MetaGeneMark (Li et al., 2014; Oh et al., 2014; Qin et al., 2014), and from the prediction results, information with length less than 100 nt (Qin et al., 2010; Nielsen et al., 2014) was filtered out. The ORF prediction results of each sample assembly were de-redundant using CD-HIT software to obtain a non-redundant initial gene catalog, using Bowtie2 to compare the Clean Data of each sample to the initial gene catalog and to calculate the number of reads of the genes in each sample. The genes supporting reads <= 2 in each sample were filtered out to obtain the final gene catalog (Unigenes) for subsequent analysis, and from the number of reads and gene lengths in the comparison reads and gene length, the abundance of each gene in each sample was calculated (Cotillard et al., 2013; Villar et al., 2015).

### 2.7. Species annotation

Using the gene catalog for comparison in the MicroNR library to obtain species annotation information for each gene (Unigene), and combined with the gene abundance tables to obtain species abundance tables for different taxonomic levels. The genes were compared with each functional database using the DIAMOND software (Buchfink et al., 2015). Unigenes were compared (blastp, evalue <= 1e-5) with Bacteria, Fungi, Archaea and Virus sequences extracted from the NCBI NR (Version: 2018.01) database (Karlsson et al., 2013).

### 2.8. Metagenomics data analysis

Functional annotation and abundance analysis of metabolic pathways in the Kyoto Encyclopedia of Genes and Genomes (KEGG) database using gene catalog data. Based on the species abundance tables at different taxonomic levels, we performed Principal Components Analysis (PCA) and Non-metric Multidimensional Scaling (NMDS) analyses, where the more similar the species composition of the samples, the closer they were in the PCA and NMDS plots. Based on the Bray–Curtis distance, Principal Co-ordinates Analysis (PCoA) analysis was performed, and the principal coordinate combination with the largest contribution rate was selected for graph display.

### 2.9. LEfSe analysis of “biomarkers” between groups

The featured microbial taxa between groups were screened using Linear discriminant analysis Effect Size (LEfSe) differential analysis. First, the rank sum test was used to detect the differential species among different groups, and Linear Discriminant Analysis (LDA) was used to achieve dimension reduction and evaluate the impact of the differential species, namely, LDA score (Segata et al., 2011) was obtained. Featured microbial taxa with LDA Score greater than the set value (LDA score = 4.0) was defined as biomarkers with statistical differences between groups.

### 2.10. Metastat analysis of functional differences between groups

In order to investigate the functions that differed significantly between groups, the functional abundance data were analyzed using the Metastats method, starting from a table of relative abundance of functions at different levels. The *p*-values were first obtained by hypothesis testing, and the *q*-values were obtained by correcting the *p*-values. Finally, the functions with significant differences were screened according to the *q*-values and box plots of the abundance distribution of the different functions between groups were drawn.

### 2.11. Annotation of resistance genes

The Comprehensive Antibiotic Resistance Database (CARD) is a new database of resistance genes that has emerged in recent years. The gene catalog is annotated with the CARD, which provides information on the distribution of resistance gene abundance and the species affiliation and resistance mechanisms of these resistance genes.

## 3. Results

### 3.1. Gut microbiota diversity in grassland small mammals

**Figure 2:**
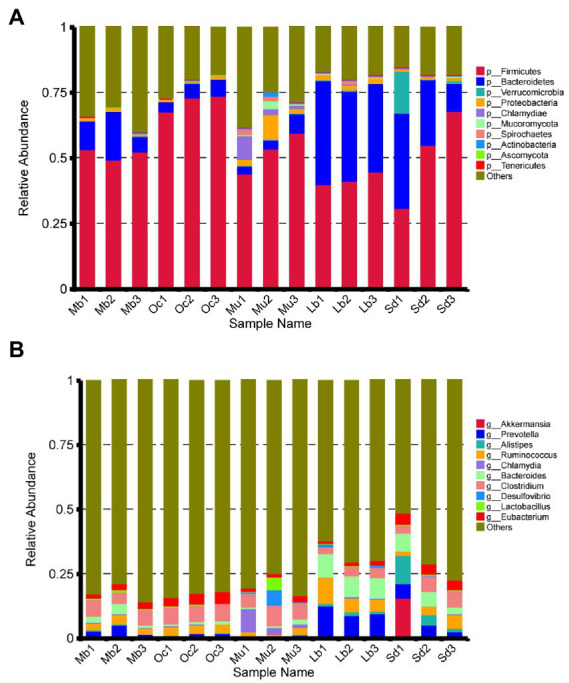
Composition of gut bacterial communities under the **(A)** phylum and **(B)** genus level among the Mb, Oc, Mu, Lb, and Sd samples.

After quality control, a total of 4,874,600 genes were obtained from the gene catalog, with 1,721,464 complete genes (both start and stop codons), representing 35.31% of the total number of genes. For gene richness characterization a sparse analysis was performed (Table 2). The estimated gene richness values were almost close to saturation in all groups, indicating that the sequencing data had sufficient coverage and that only a very small number of genes may have been undetected. In this study, a total of 233,591 unique genes were identified in the cecum feces of the plateau zokor (Supplementary Figure S1A). Furthermore, we analyzed the gut microbiota composition of plateau zokors under the phylum and genus levels. The top 10 microbial taxa in terms of maximum relative abundance in the gut microbiota of each sample are shown in Figure 2A, with Firmicutes and Bacteroidetes being the predominant phylum in all sites. Inter-group analyses revealed that the Firmicutes (51.87%) and the Bacteroidetes (11.94%) were found under phylum levels in the gut of plateau zokors, respectively (Supplementary Table S1). Figure 2B shows the composition of the gut microbiota under the genus level, with *Clostridium* (5.57%), *Prevotella* (3.54%), *Ruminococcus* (2.92%), *Bacteroides* (2.56%), *Eubacterium* (2.36%,) *Alistipes* (0.26%), *Lactobacillus* (0.26%), *Desulfovibrio* (0.21%), and *Chlamydia* (0.13%) in the plateau zokors. The heat map of gene number and abundance clustering under the species level is shown in Supplementary Figure S2.

**Table 2:** Statistical table of gene catalog basic information.

ORFs no.	4,874,600
Integrity: end	1,119,013 (22.96%)
Integrity: all	1,721,464 (35.31%)
Integrity: none	606,862 (12.45%)
Integrity: start	1,427,261 (29.28%)
Total Len. (Mbp)	2,930.47
Average Len. (bp)	601.17
GC percent	47.75

### 3.2. Differences in the composition of gut microbial in plateau zokor

**Figure 3:**
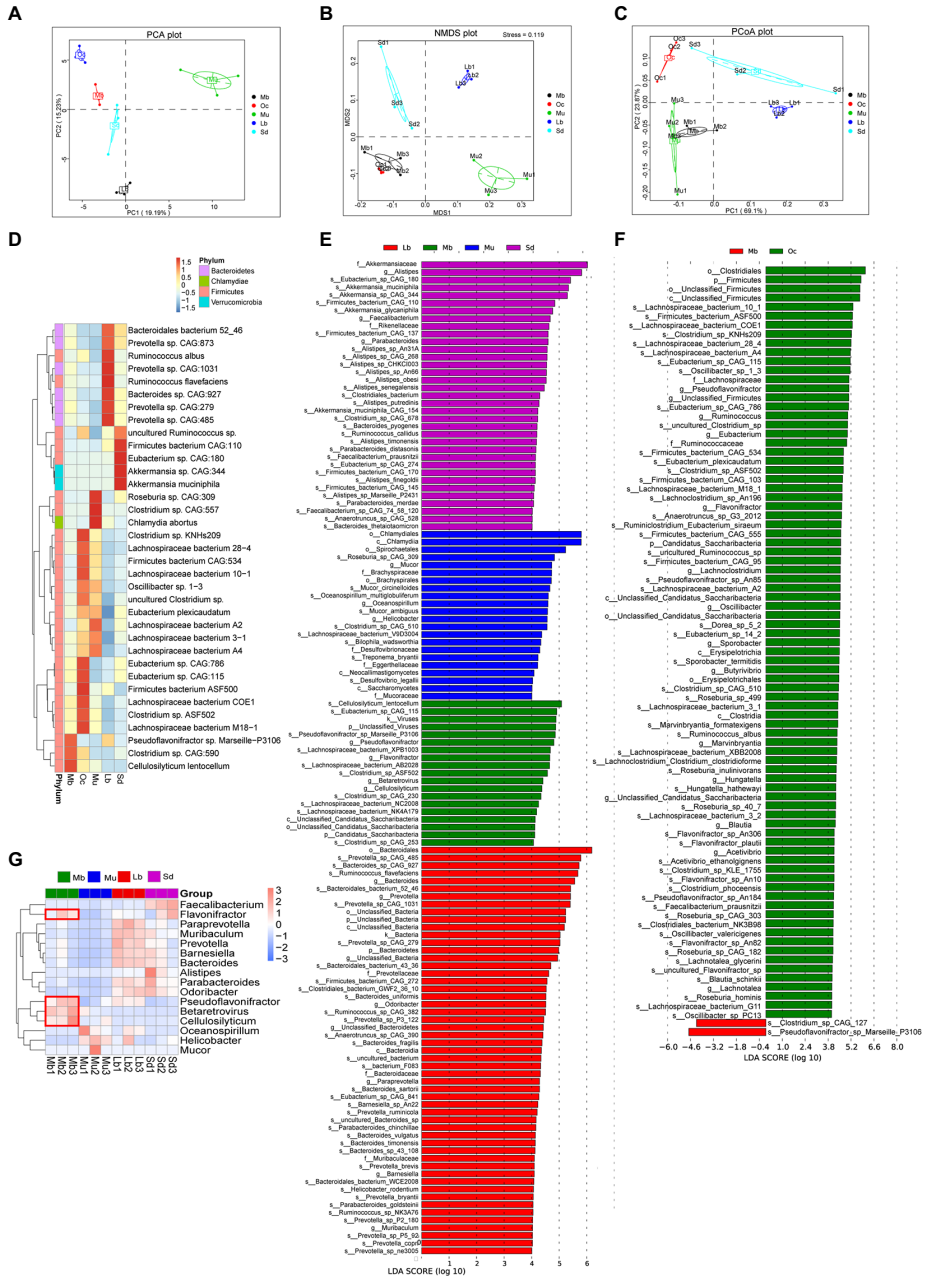
Analysis of gut microbiota with significant differences. **(A)** PCA analysis among the Mb, Oc, Mu, Lb, and Sd groups under phyla level. The horizontal coordinates indicate one principal component, vertical coordinates indicate the second principal component, percentages indicate the contribution of the principal component to sample variation, each point in the figure represents a sample, and the samples are represented by the same color. **(B)** NMDS analysis among the Mb, Oc, Mu, Lb, and Sd groups under phyla level (Stress = 0.119). Each point in the graph represents a sample, and the distance between points represents the degree of difference. When the Stress is less than 0.2, it indicates that the NMDS analysis has certain reliability. **(C)** PCoA analysis among the Mb, Oc, Mu, Lb, and Sd groups under phyla level The horizontal coordinate indicates one principal component, the vertical coordinate indicates another principal component, and the percentage indicates the contribution of the principal component to the variance of the samples. **(D)** Relative abundances of bacterial species among the Mb, Oc, Mu, Lb, and Sd groups. **(E)** The featured microbial taxa of each animal species according to the results of LEfSe differential analysis. The bar chart of the distribution of LDA score shows microbial taxa with an LDA Score greater than a set value (LDA score > 4.0), i.e., Biomarkers that are statistically different between the Mb, Mu, Lb, and Sd groups, and the length of the bar chart represents the size of the effect of the differential species. **(F)** The featured microbial taxa of each animal species according to the results of LEfSe differential analysis. The differential analysis was conducted between the Mb and Oc groups (LDA score > 4.0). **(G)** Relative abundances of bacterial taxa (Biomarker with statistical differences) varied across the Mb, Mu, Lb, and Sd samples according to the results of LEfSe (LDA score > 4.0).

Specific bacterial flora may influence the adaptation of the plateau zokor to the living environment, and we continued to look for “biomarkers” with statistical differences.Firstly, by using the method of PCA, NMDS, and PCoA under the phylum level, the results showed significant differences in the gut microbial composition of the five groups (Figures 3A–C). The results of the interspecific relative thermogram analysis among the Mb, Oc, Mu, Lb, and Sd groups (Figure 3D) showed that the relative abundance of three species of bacteria was high in the gut microbial of plateau zokors, namely *Pseudoflavonifractor* sp., *Marseille*-P3106, *Clostridium* sp. CAG:590, and *Cellulosilyticum lentocellum*. In order to better explore the adaptation mechanisms of plateau zokors in the plateau region, Biomarker was used to screen for species with significant differences between groups using LEfSe analysis. In order to distinguish which biomarker with statistical differences are unique to plateau zokors and which may be common to plateau species, we did not directly choose the all five groups to LEfSe analysis (LDA score > 4.0), but instead took an indirect comparative approach, specifically using these two plateau species separately from the other three species (Lb, Mu, and Sd groups) for the differential analysis, selecting the featured microbial taxa that are present in both plateau species (Figure 3E; Supplementary Figure S3). The two plateau species (Mb and Oc groups) were then chosen to LEfSe analysis, from which the featured microbial taxa unique to plateau zokors could be obtained. We found the following bacteria were enriched in both plateau animal species, *Clostridium* sp. CAG 253, *Clostridium* sp. ASF502, *Clostridium* sp. CAG 230, *Cellulosilyticum lentocellum*, *Lachnospiraceae bacterium* AB2028, *Lachnospiraceae bacterium* NK4A179, *Eubacterium* sp. CAG 115, *Pseudoflavonifractor* genus, *Flavonifractor* genus, *Candidatus saccharibacteria*, *Betaretrovirus* genus. In addition to this, there are also significant abundance differences among *Pseudoflavonifractor* sp. Marseille-P3106, *Lachnospiraceae bacterium* XPB1003, *Lachnospiraceae bacterium NC2008*, viruses kingdom, and unclassified_viruses. LEfSe analysis (LDA score > 4.0) was then carried out for plateau zokors and plateau pikas and the distribution of LDA score for the differential species is shown that (Figure 3F) the *Clostridium* sp. CAG 127and the *Pseudoflavonifractor* sp. Marseille P3106 may be the unique Biomarker to plateau zokors.

In order to investigate the abundance of statistically different Biomarkers among different groups, *p*-values were obtained by hypothesis testing using the Metastats method, and *q*-values were obtained by correcting the *p*-values. As shown in Figure 3G, the featured microbial taxa of each animal species were *Flavonifractor*, *Pseudoflavonifractor*, *Cellulosilyticum*, and *Betaretrovirus*.

### 3.3. The metabolic function of gut microbial facilitates adaptation of the plateau zokor to the plateau environment

**Figure 4:**
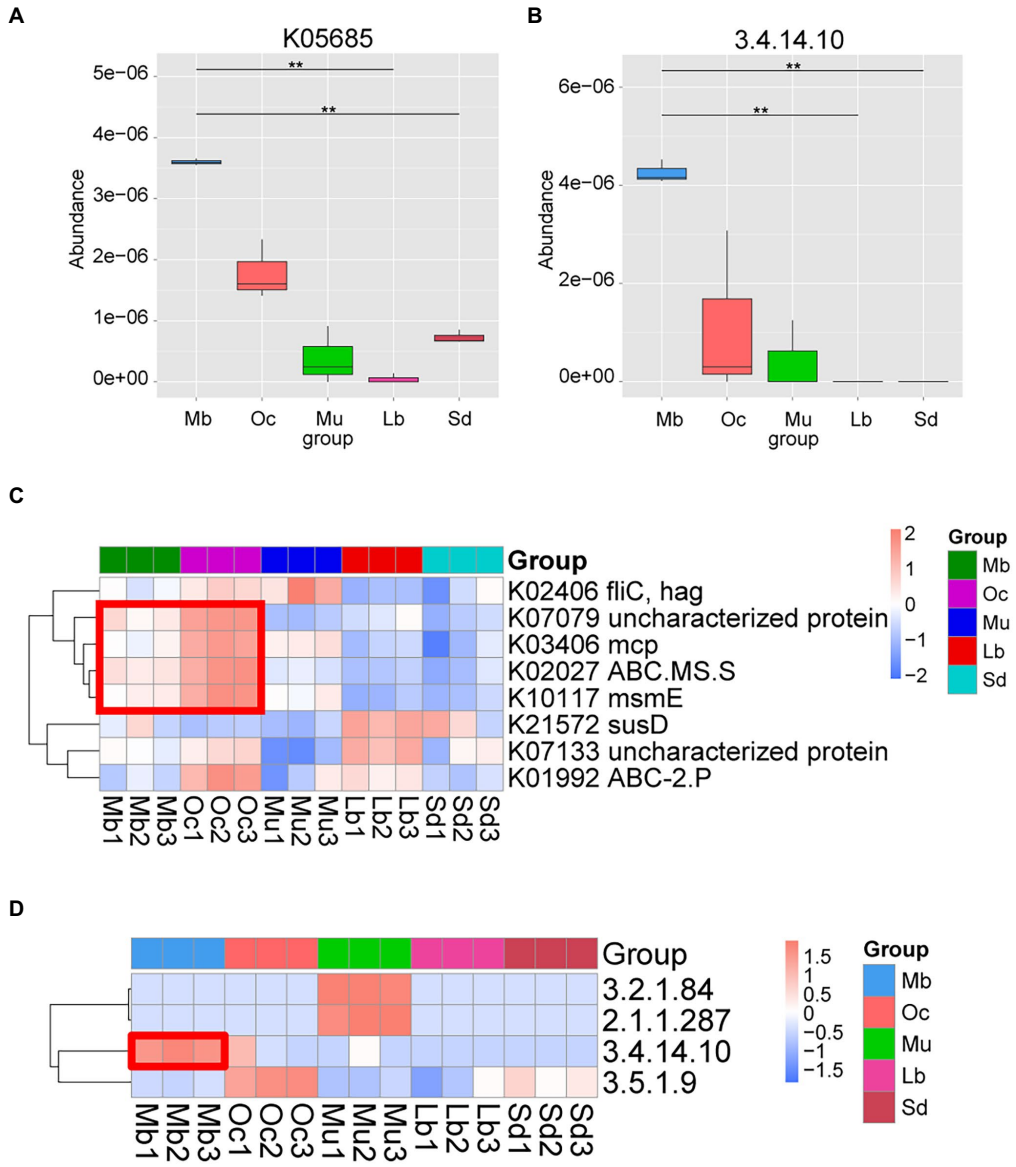
Functional metagenomic comparison of the gut microbiota in different groups. **(A)** KO terms with significant differences between groups. The horizontal axis is the group name and the vertical axis is the relative abundance of the corresponding species. Pairwise statistical analysis was done by Metastats. * and ** denote *q*-value < 0.05 and *q*-value<0.01, respectively. **(B)** EC Number with significant differences between groups according to the results of Pairwise statistical analysis by Metastats. **(C)** Heatmap of KEGG ortholog pathways showing different enrichments according to the results of LEfSe differential analysis (LDA score > 3.0). **(D)** Heatmap of KEGG EC Number showing different enrichments according to the results of Pairwise statistical analysis by Metastats.

In order to better understand the functional differences in the gut microbiota of plateau zokors, DIAMOND software was used to compare Unigenes with various functional databases, and the relative abundance of different functional levels was calculated from the comparison results. After Metastats analysis using the KEGG, it was found that the metabolic functions of the samples from different geographic regions were statistically significantly different. We found that the Organismal Systems: Sensory System metabolic pathway has obvious abundance enrichment in plateau zokors (Supplementary Figure S4). The difference analysis of K05685 pathway (Figure 4A) and E.C found that 3.4.14.10 tripeptidyl peptidase II (TPPII) (Figures 4B,D) was also significantly enriched in plateau zokor. In addition, we performed LEfSe tests to detect KEGG pathways based on significantly different abundances between groups. According to the results of the LEFSe test, K07079, K03406, K02027, K10117 were significantly enriched in both plateau zokors and plateau pikas (Figure 4C).

### 3.4. Resistance gene analysis

The CARD resistance gene database was used to align the gene sequences to annotate the resistance genes. The core component of the database is the Antibiotic Resistance Ontology (ARO), which integrates information on sequences, antibiotic resistance, mechanisms of action, and associations between AROs, and provides an online interface between AROs and databases such as PDB and NCBI. The results showed that the genetic resistance of the animal host in the plateau region differed significantly from those in other grassland regions (Supplementary Figure S5C), and the types and absolute abundance of resistance genes were lower than those in other regions (Supplementary Figures S5A,B). The resistance genes of plateau zokors and plateau pika were mainly APH6-Ic, vanF, MexS, vanTN and LRA-13 (Supplementary Figure S5D).

## 4. Discussion

The plateau zokor, a typical subterranean rodent inhabiting the Tibetan plateau, has to cope with the complex environment of high humidity, limited oxygen, high CO_2_ concentration, low temperature and food scarcity (Shao et al., 2015; Su et al., 2015), and there have been studies on its adaptive evolution (Pu et al., 2019b; Zhang T. et al., 2021). However, compared with the study on the adaptation of the plateau pika, less study has been done on plateau zokor in terms of the unique lifestyle (Li et al., 2018). Considering the similarity of gut microbiota among sympatric species, we first chose plateau pika as a sympatric control species when conducting the study. Then the animal host species that is most suited to the local grassland environment was selected as a control species in other altitudinal regions, so that it was easier to find gut microbiota with the ability to significantly facilitate the adaptation of the host to the local environment. Therefore, based on the results of field sampling, we selected Mongolian gerbil, and Daurian ground squirrel from other regions as control species.

The first problem that plateau zokors have to overcome when faced with the harsh living conditions is energy intake, as the low oxygen levels and extreme cold weather at high altitudes require more energy intake to maintain the animal’s body temperature (Qiu et al., 2012; Wang et al., 2021; Du et al., 2022).

In this study, we discovered that the abundance of unigenes in the gut microbiota of plateau zokors and plateau pikas was higher than that of the other three grassland small mammals, and that a more varied gut microbiota might more effectively control energy metabolism (Wang et al., 2020). For example, the gut microbiota of brown bear with higher diversity may regulate energy metabolism and promote fat storage, whereas less diverse gut flora may slow host metabolism (Sommer et al., 2016). Compared with low-altitude mammals, the relative abundance of Firmicutes and Bacteroidetes in the gut microbiota plateau zokor and plateau pika of high-altitude was higher. For example, in this study, a large amount of *Cellulosilyticum lentocellum* was enriched in plateau zokor and plateau pika in the Qinghai-Tibet Plateau, which has cellulolytic properties and can hydrolyze cellulose and xylan (van der Wielen et al., 2002; Cai and Dong, 2010; Baba et al., 2019). We hypothesize that this may be related to the fact that microbial communities in plateau species may have a higher ability to utilize high fiber forage to help them meet their energy requirements in cold and high altitude habitats, which may help the host maintain gut homeostasis, energy homeostasis, and core body temperature in harsh environments (Liu et al., 2021). The concept that hypoxia induces inflammation has been generally accepted from studies of hypoxic signaling pathways (Eltzschig and Carmeliet, 2011), so plateau zokors also have to cope with the inflammatory response caused by low pressure, hypoxia and high concentrations of CO_2_ in the burrow. Vascular leakage, accumulation of inflammatory cells in multiple organs and elevated serum cytokine levels have been shown to occur in humans and mice following short-term exposure to low oxygen concentrations (Thompson et al., 2004; Eltzschig et al., 2005). High concentrations of CO_2_ in the environment can also cause tissue damage in the body and produce an inflammatory response (Thom et al., 2017; da Costa and Val, 2020). Plant-based fiber intake has been shown to increase microbiota diversity and reduce markers of inflammation (Ma et al., 2021; Wastyk et al., 2021). Additionally, gut microbes produce metabolites such as short-chain fatty acids (SCFAs), volatile fatty acids (VFA), essential amino acids and vitamins through their collective metabolic activities, which contribute to host to evolve adaptations (Nicholson et al., 2012; Mukherjee et al., 2020). For example, butyrate has been shown to have several beneficial effects, including being an excellent nutrient for epithelial cells, and having immunomodulatory and anti-inflammatory properties (Meehan and Beiko, 2014; Berger et al., 2021). Current studies have shown that SCFAs inhibit inflammation mainly by inhibiting the NF-κB pathway and/or histone deacetylase function (HDACi), thereby downregulating pro-inflammatory cytokines (Zhang et al., 2019). In this study, both plateau species were found to be enriched with a large number of SCFAs-producing strains. For example, both plateau zokors and plateau pikas were enriched with *Lachnospiraceae*. In addition, plateau zokors were enriched with *Lachnospiraceae bacterium* XPB1003 and *Lachnospiraceae bacterium* NC2008 alone. Furthermore, the enrichment of *Clostridium* spp. in plateau zokors may not only enable these hosts to obtain more energy from their food (Ma et al., 2019), but also convert dietary fiber into SCFAs such as butyric acid (Schwiertz et al., 2010; West et al., 2019). *Flavonifractor* spp. can also produce SCFAs (Chen et al., 2020; Gao et al., 2021). Studies have shown that *Flavonifractor plautii* can exhibit lower levels of inflammation and that the active component of FP’s lipoteichoic acid mediates strong inhibition of interleukin (IL)-17 signaling (Mikami et al., 2020). *Eubacterium* spp. provides butyrate-mediated protection and is considered a new generation of “potentially beneficial microorganisms” whose presence in the gut is largely associated with increased dietary fiber intake (Kanauchi et al., 2006; Duncan et al., 2007; Vermeiren et al., 2012; Geirnaert et al., 2017). *Pseudoflavonifractor* is also a bacterium that can produce butyrate (Kläring et al., 2013; Sakamoto et al., 2018). At present, there are few studies on *Candidatus saccharibacteria*, which are directly involved in the degradation of hydrocarbons (Figueroa-Gonzalez et al., 2020; Nie et al., 2022). Similar phenomena have also been found in other plateau species. For example, a study used a multi-omics approach to examine fecal samples from high-and low-altitude humans and pigs, and direct evidence of consistent results linking genes to metabolites suggests that gut microbiota from high-altitude Tibetan pigs may produce more short-or long-chain fatty acids (Zeng et al., 2020). These evidences indicate that the plateau zokor has a large number of bacteria that decompose dietary fiber and produce SCFAs, which may facilitate the adaptation of plateau zokor to the plateau environment.

In this study, we used KEGG database to predict the function of Metagenomic data of plateau zokors. Our results suggest that the estimated gene functional profile of the microbiome is significantly influenced by altitude. Some metabolic pathways were significantly enriched in both plateau zokors and plateau pikas in the Qinghai-Tibet Plateau region. Most strikingly, those genes involved in Organismal Systems: Sensory system in the KEGG level 2 pathway were enhanced in plateau zokors. This might be due to plateau zokors’ protracted burrowing habits, which maximize their capacity to assist them in perceiving environmental changes in the face of degraded vision. K05685, K10117, and K02027 are all involved in catalytic carbohydrate uptake and directly involved in ATP production (Schneider, 2001; Webb Alexander et al., 2008; Modali and Zgurskaya, 2011; Cerisy et al., 2019). K03406 is involved in bacterial chemotaxis, which is essential for host colonization and virulence of many pathogenic bacteria causing human, animal and plant diseases (Wadhams and Armitage, 2004; Rosenberg et al., 2007; Salah Ud-Din and Roujeinikova, 2017). The function of the K07079 signaling pathway is unknown, and it may also play a role in promoting host adaptation, which needs to be further verified by experiments.

In high-altitude environments, low oxygen and high UV radiation may lead to DNA and protein damage, while genes associated with replication and repair may help to reduce damage to biomolecules (Dosek et al., 2007; Sinha et al., 2010). In addition, the harsh environmental stress on the Tibetan plateau can also put the organism in a state of oxidative stress (Cui et al., 2016). As altitude increases in mountainous areas, the production of reactive oxygen species (ROS) accelerates, which may lead to severe oxidative tissue damage, capable of damaging proteins, nucleic acids, polysaccharides and lipids, thereby inducing apoptosis (Strapazzon et al., 2016; Debevec et al., 2017; Mrakic-Sposta et al., 2022). More importantly, our KEGG analysis results show that 3.4.14.10 tripeptidyl peptidase II (TPPII) is significantly enriched in the intestine of plateau zokors. TPPII has demonstrated independent enzymatic activity involved in a wide range of activities, including antigen processing, apoptosis, DNA damage repair and cell division (Rockel et al., 2012; Tan et al., 2016). Studies have shown that in several malignant cell lines, TPPII translocates into the nucleus after γ-irradiation and ROS production and is involved in DNA repair (Preta et al., 2009, 2010). Involvement in antigen processing is probably the most studied aspect of the potential physiological role of TPP II (Tomkinson, 2019). TPPII is involved in cancer and antigen processing by MHC-I presentation and the presented antigens can be detected by CD8+ T cells, a process that is essential for the detection and destruction of cancer cells or cells infected by viruses (Seifert et al., 2003; Diekmann et al., 2009; Embgenbroich and Burgdorf, 2018). Although there is still controversy over antigen processing, TPPII has been shown to be associated with immunodeficiency, autoimmunity and neurodevelopmental retardation (Stepensky et al., 2015), implying that TPPII may be crucial to the immune system of plateau zokors. Therefore, we hypothesize that TPPII may contribute to the adaptation of plateau zokors to survive in confined burrows at high altitudes. However, our results are based on predicted metagenomics only and may not accurately reflect how TPPII functions in plateau zokors. Further studies should be conducted for experimental validation to explore the role of TPPII in the environmental adaptation of plateau zokors.

In addition, the complex topography and physical barriers of the Tibetan plateau not only significantly reduce the dispersal of organisms, but may affect patterns of gene flow, which could ultimately affect the current spatial distribution of endemic plateau species and their genetic diversity (Hewitt, 2000; Toju, 2008). Compared with some ground-moving rodents, the plateau zokor has a lower activity time and frequency (Kang et al., 2020). The unique geographical location and habits of plateau zokors also result in a low probability of anthropogenic and pathogenic infestation, with the potential for a large number of unknown pathogenic microorganisms within the population (Cao et al., 2014; Zhao et al., 2014). As the largest population of mammals, rodents transmit a variety of infectious zoonotic diseases to humans (Morand et al., 2015). Our results suggest that plateau zokors have a large number of unknown viruses (Supplementary Figure S1B), and maybe a potential source of zoonotic transmission, increasing the risk of pathogenic spillover. Furthermore, resistance genes are widespread in the environment and increased antibiotic consumption directly leads to environmental pollution by antibiotics, which can threaten the balance of ecosystems and human health (Polianciuc et al., 2020). Although the abundance of resistance genes in plateau zokors was lower and the species varied significantly compared to other animal species, it also indicated that resistance genes are prevalent in the Tibetan plateau region. Therefore, it is necessary to increase active surveillance and reverse pathogenic research on special wildlife in the Tibetan Plateau region, to explore the pathogenic mechanisms of newly present pathogens, assess the risk of disease outbreaks and to provide early warning.

## 5. Conclusion

Different geographical environments have significant effects on the composition of mammalian gut microbiota, while at the same time the gut microbiota can facilitate adaptation of a variety of small mammals to different geographical environments. Our study demonstrates that the gut microbiota of plateau zokors is highly enriched with the phylum Firmicutes and Bacteroidetes, among which *Cellulosilyticum lentocellum* and other bacteria play a role in the breakdown of dietary fiber. This, along with the enrichment of some signaling pathways related to energy uptake, ensure the energy supplementation of the plateau zokor in a low oxygen and low-pressure environment. The gut microbiota enriched with *Lachnospiraceae*, *Pseudoflavonifractor*, *Eubacterium, Flavonifractor*, *Clostridium*, and other flora, which produce SCFAs, may help plateau zokors to reduce the inflammatory response caused by low pressure, hypoxia, and high concentrations of CO_2_ in the burrow as they adapt to the high-altitude environment. The gut microbial metabolite TPPII may help plateau zokors adapt to the tissue damage and immune response caused by the high-altitude confined burrow environment. These unique gut microbiota and metabolites together contribute to the adaptation of plateau zokors to the plateau environment.

## Data availability statement

The data presented in the study are deposited in the National Genomics Data Center (NGDC), accession number PRJCA014883.

## Ethics statement

The animal study was reviewed and approved by this study was conducted in accordance with the Guidelines for the Care and Use of Animals in Research published by the Institute of Zoology, Chinese Academy of Sciences. This study was reviewed and approved by the Animal Ethics Committee of the Institute of Zoology, Chinese Academy of Sciences (2019FY100300-03).

## Author contributions

HH and BH contributed to the conception of the study. JW, YL, JP, GL, YoH, and HZ contributed significantly to collect samples and perform the experiment. JG, YX, and SH contributed significantly to analysis and manuscript preparation. BW and YaH helped to perform the analysis with constructive discussions. BH, YL, and JW performed the data analyses and wrote the manuscript. All authors contributed to the article and approved the submitted version.

## Funding

This study was supported by the National Key Research and Development Program of China (No. 2022YFC2601602), the Major Program of National Natural Science Foundation of China (No. 32090023), and National Forestry and Grassland Administration, China.

## Conflict of interest

The authors declare that the research was conducted in the absence of any commercial or financial relationships that could be construed as a potential conflict of interest.

## Publisher’s note

All claims expressed in this article are solely those of the authors and do not necessarily represent those of their affiliated organizations, or those of the publisher, the editors and the reviewers. Any product that may be evaluated in this article, or claim that may be made by its manufacturer, is not guaranteed or endorsed by the publisher.
