# Supplementary material for: Gut microbiota facilitates adaptation of the plateau zokor (Myospalax baileyi) to the plateau living environment
Source: Front Microbiol. 2023 Feb 24;14:1136845. doi: 10.3389/fmicb.2023.1136845 (PMC9998695; doi:10.3389/fmicb.2023.1136845)
Supplement: Supplementary file 1 [file Table_1.DOCX]

Supplementary Material

# Supplementary Figures and Tables

## Supplementary Figures

**The original images are in the same folder**


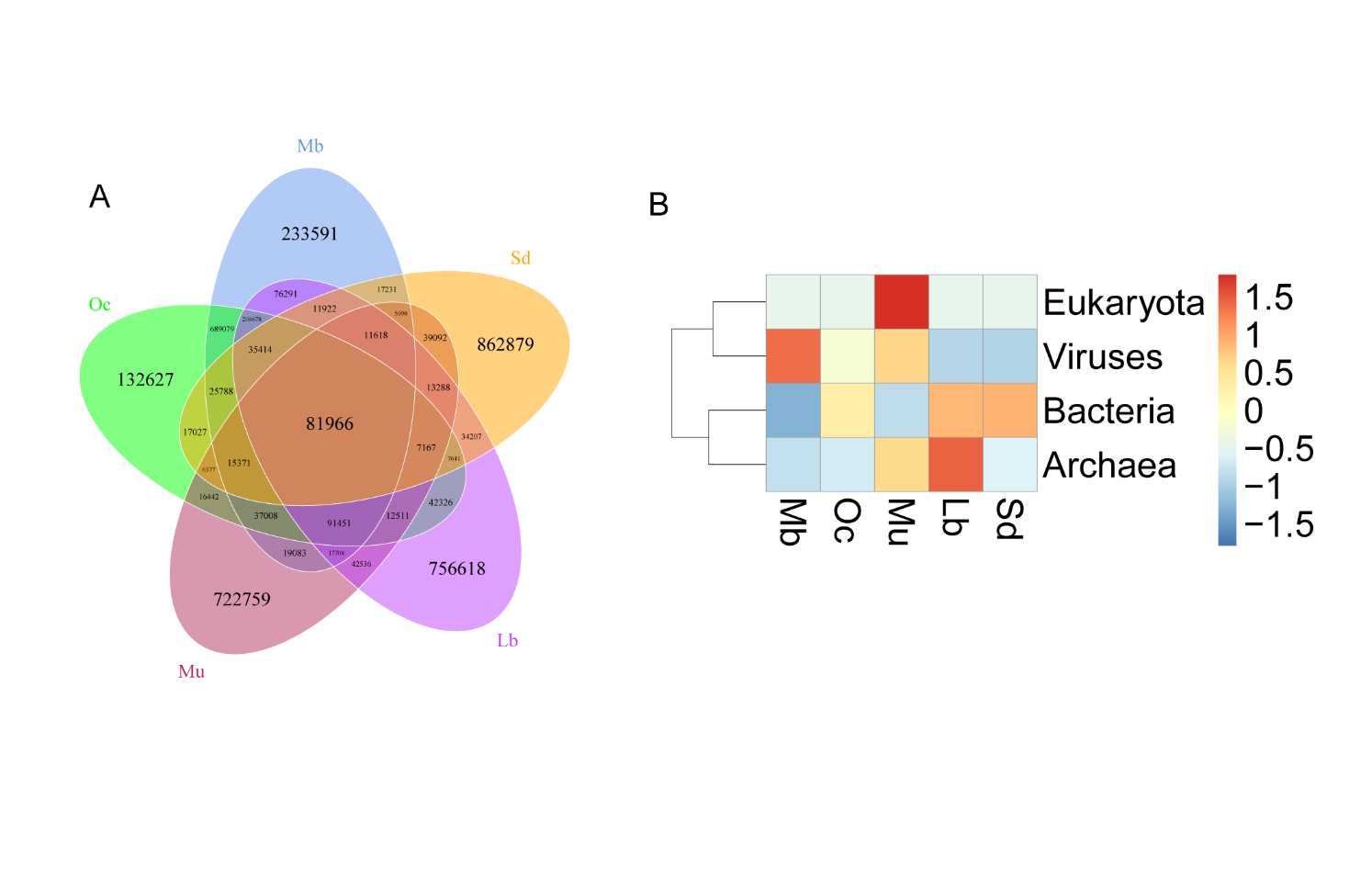


**Supplementary Figure 1.** Gene annotation information **(A)** Venn diagram analysis of gene numbers. **(B)** Heat map of relative abundance clustering under the kingdom level between groups.


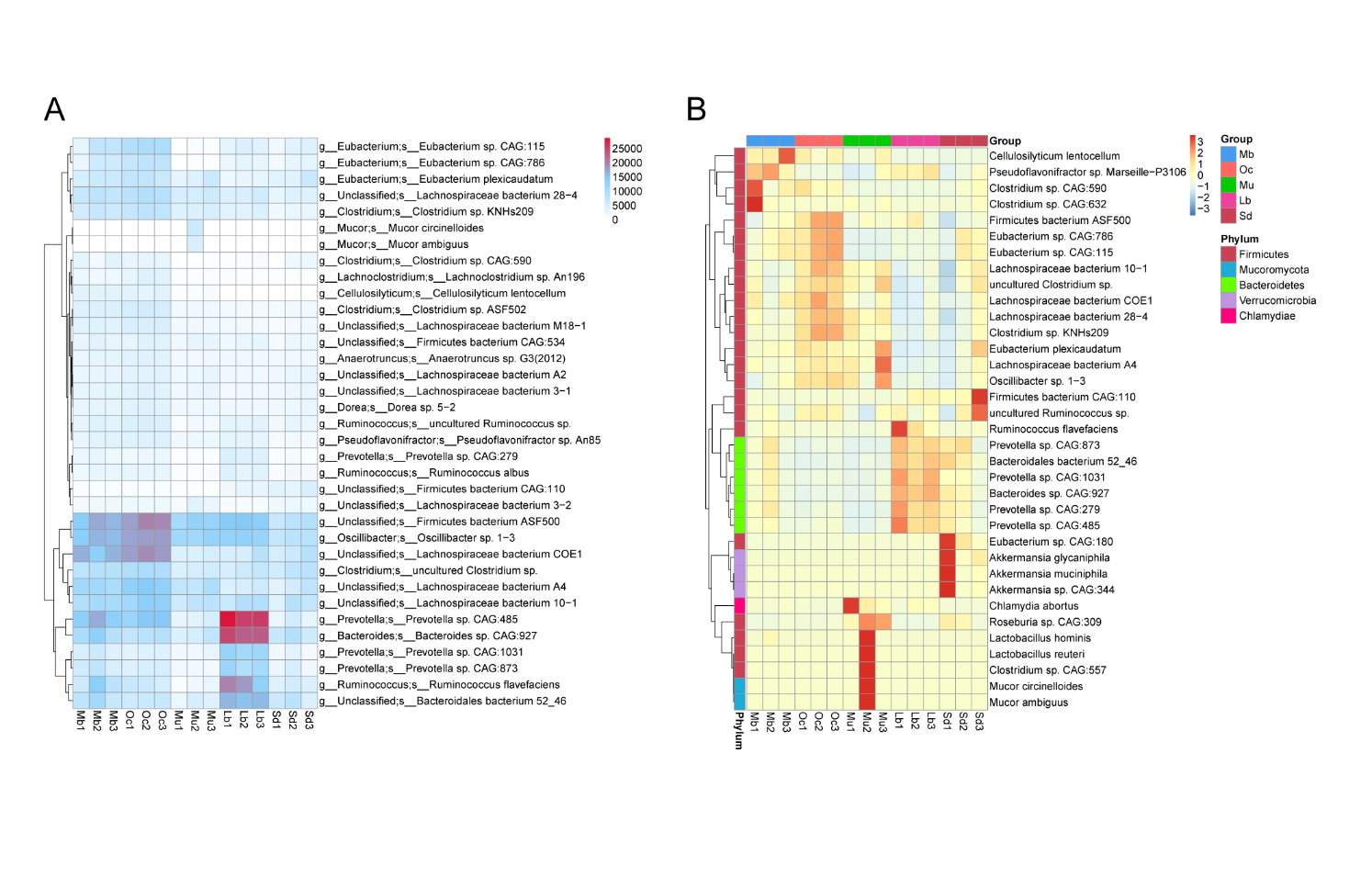


**Supplementary Figure 2.** Heat map of the number and abundance of genes clustered under the species level **(A)** Heat map of the number and abundance of genes clustered under the species level. **(B)** Heat map of relative abundance clustering under species level.


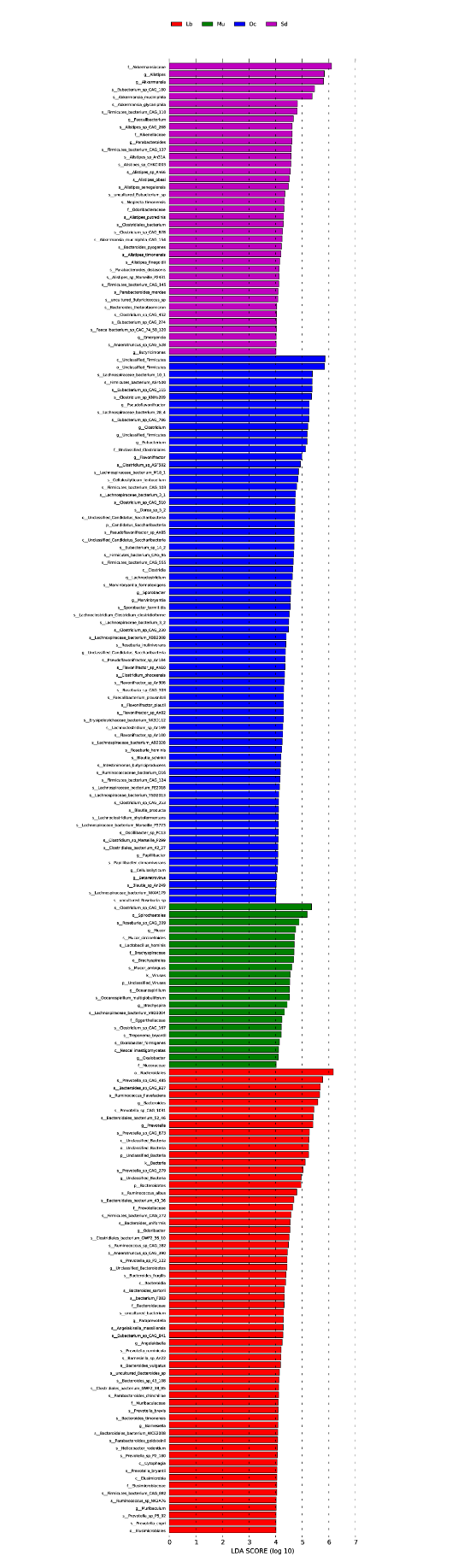


**Supplementary Figure3.** The featured microbial taxa according to the results of LEfSe among the Oc, Mu, Lb, and Sd groups (LDA score > 4.0).

**
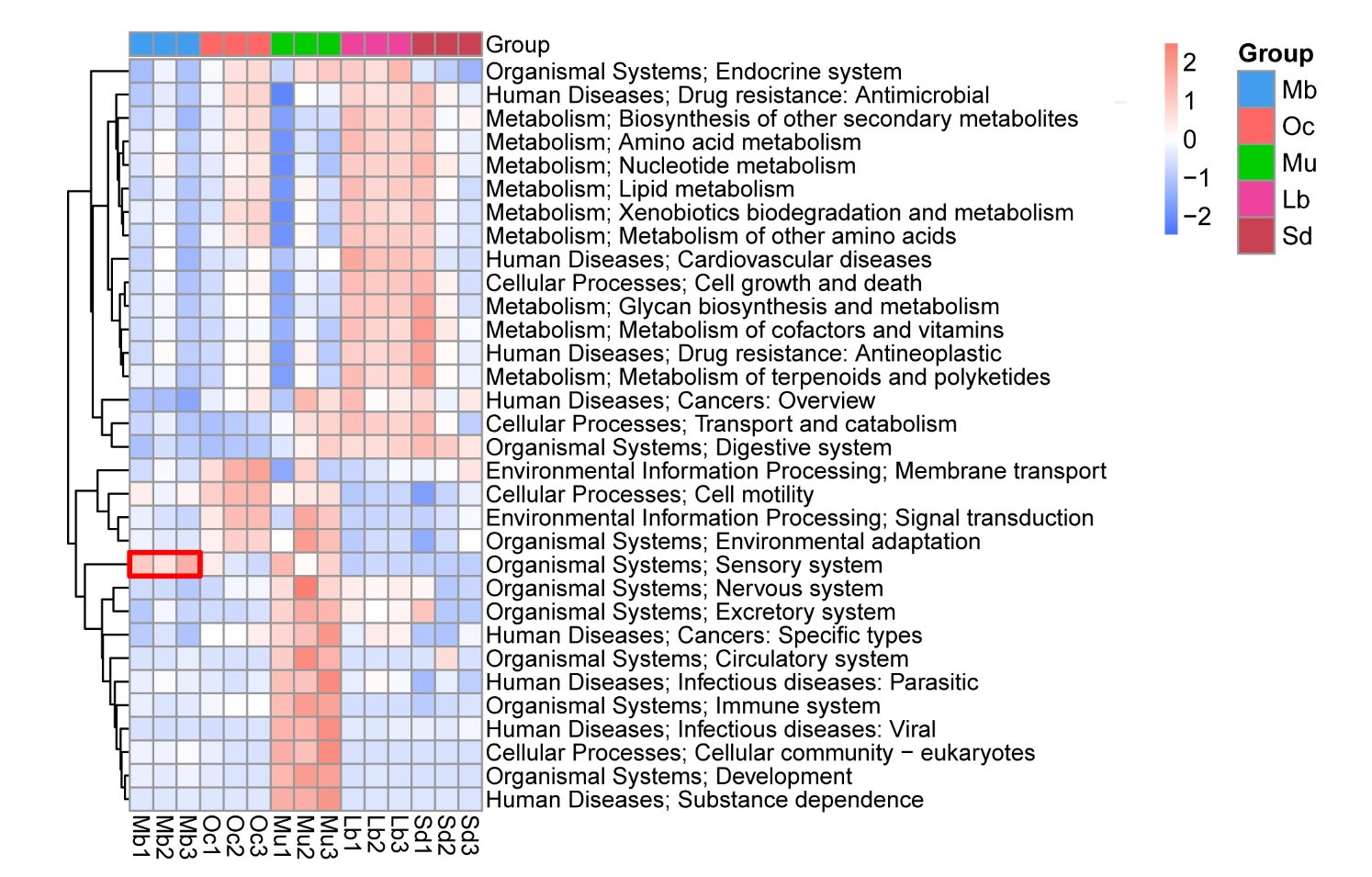
**

**Supplementary Figure 4.** Relative abundance of differentially functional KEGG level2 metabolic pathways according to the MetaStats analysis.

**
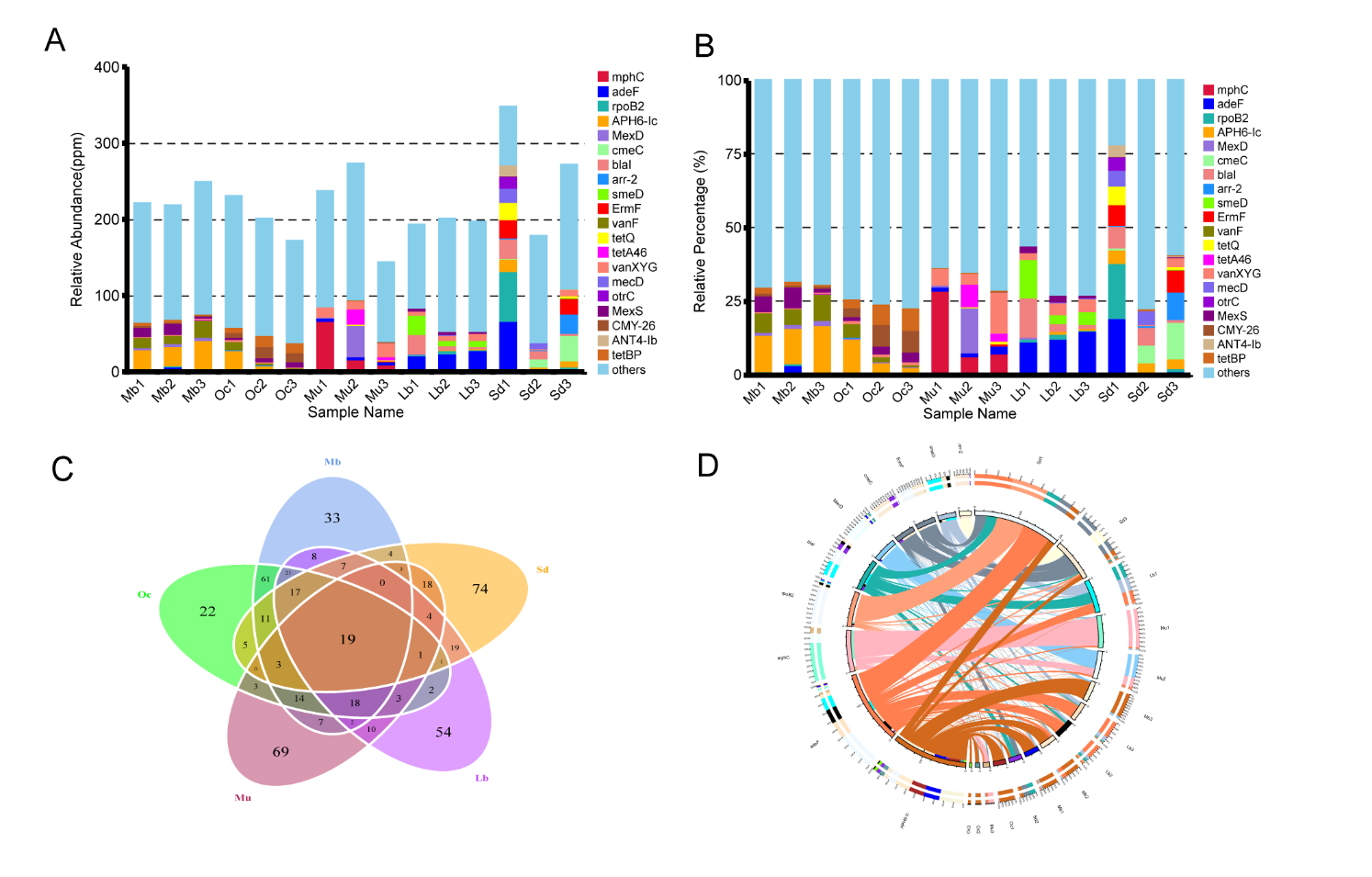
**

**Supplementary Figure 5.** Abundance histogram of different AROs in each sample **(A)** The Relative abundance of all genes in each sample by ARO. **(B)** The Relative abundance of top20 AROs in all AROs, others is the sum of relative abundances of non-top20 AROs. **(C)** Venn diagram analysis of the number of resistance genes between groups. **(D)** Overview circle diagram of the resistance gene, with sample information on the right and ARO information on the left.

| phylum | Firmicutes | Bacteroidetes | Verrucomicrobia | Proteobacteria | Chlamydiae | Mucoromycota | Spirochaetes | Actinobacteria | Ascomycota | Tenericutes | Others |
| --- | --- | --- | --- | --- | --- | --- | --- | --- | --- | --- | --- |
| Mb | 0.518736 | 0.119407 | 0.00023 | 0.009613 | 0.001345 | 0.000033 | 0.000956 | 0.002116 | 0.000016 | 0.001278 | 0.34627 |
| Oc | 0.714612 | 0.05673 | 0.000139 | 0.008082 | 0.000783 | 0.000016 | 0.001129 | 0.002621 | 0.000012 | 0.000809 | 0.215067 |
| Mu | 0.525598 | 0.047292 | 0.000763 | 0.046048 | 0.042487 | 0.010656 | 0.016126 | 0.006097 | 0.002014 | 0.001206 | 0.301713 |
| Lb | 0.421601 | 0.360694 | 0.000607 | 0.020382 | 0.003825 | 0.000013 | 0.007775 | 0.002325 | 0.000027 | 0.0015 | 0.181251 |
| Sd | 0.515144 | 0.239748 | 0.057224 | 0.009982 | 0.000506 | 0.000047 | 0.001087 | 0.005503 | 0.000039 | 0.001779 | 0.168941 |

**Supplementary Table 1** The top 10 Gut microbiota between group at the phylum level

**Note:** Data is the mean of the samples within the group
