# Supplementary figures and images for: Gut microbiota facilitates adaptation of the plateau zokor (Myospalax baileyi) to the plateau living environment
Source: Front Microbiol. 2023 Feb 24;14:1136845. doi: 10.3389/fmicb.2023.1136845 (PMC9998695; doi:10.3389/fmicb.2023.1136845)

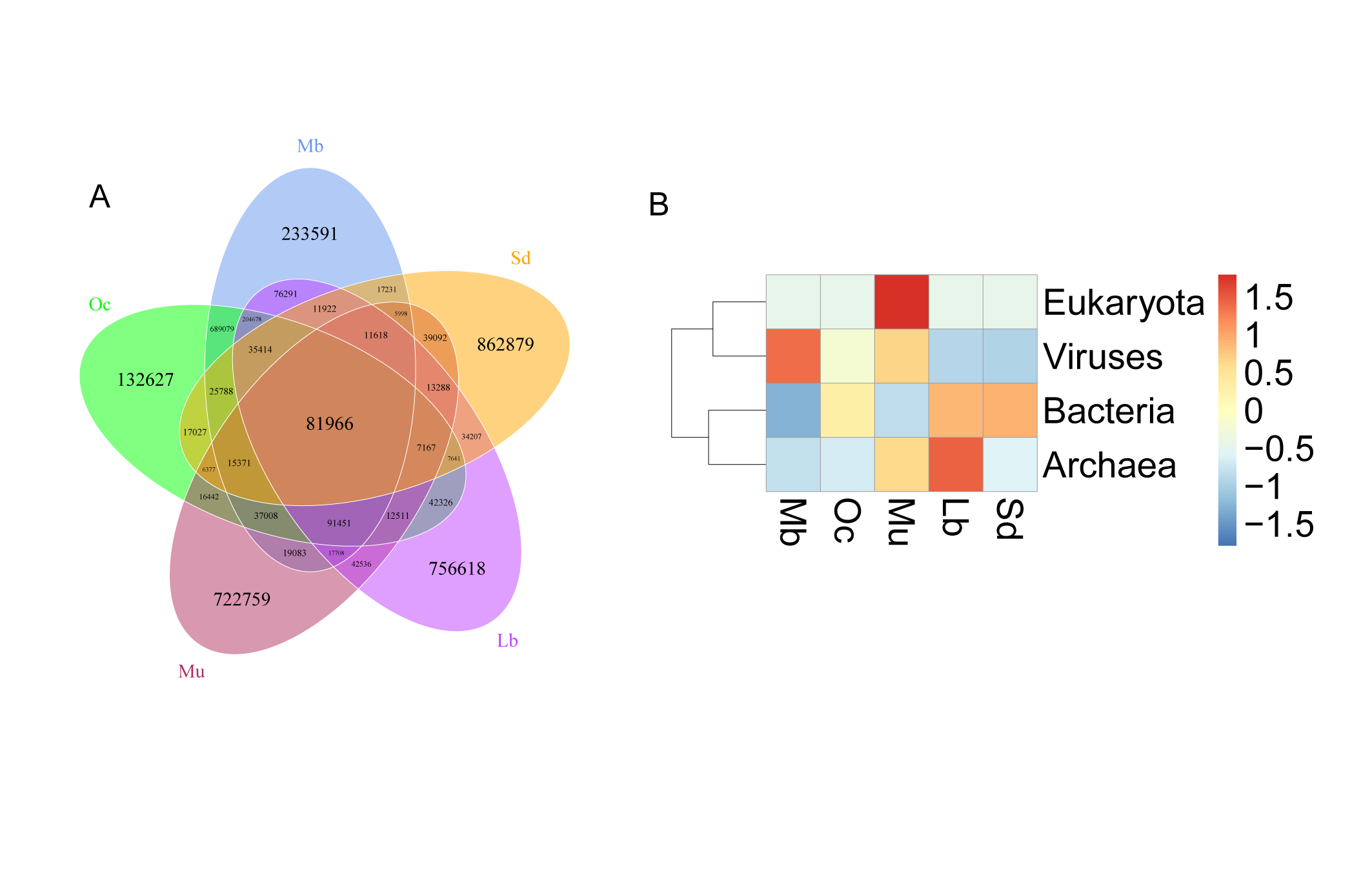

Supplement: Supplementary file 2 [file Data_Sheet_1.ZIP › Figure S1.tif]

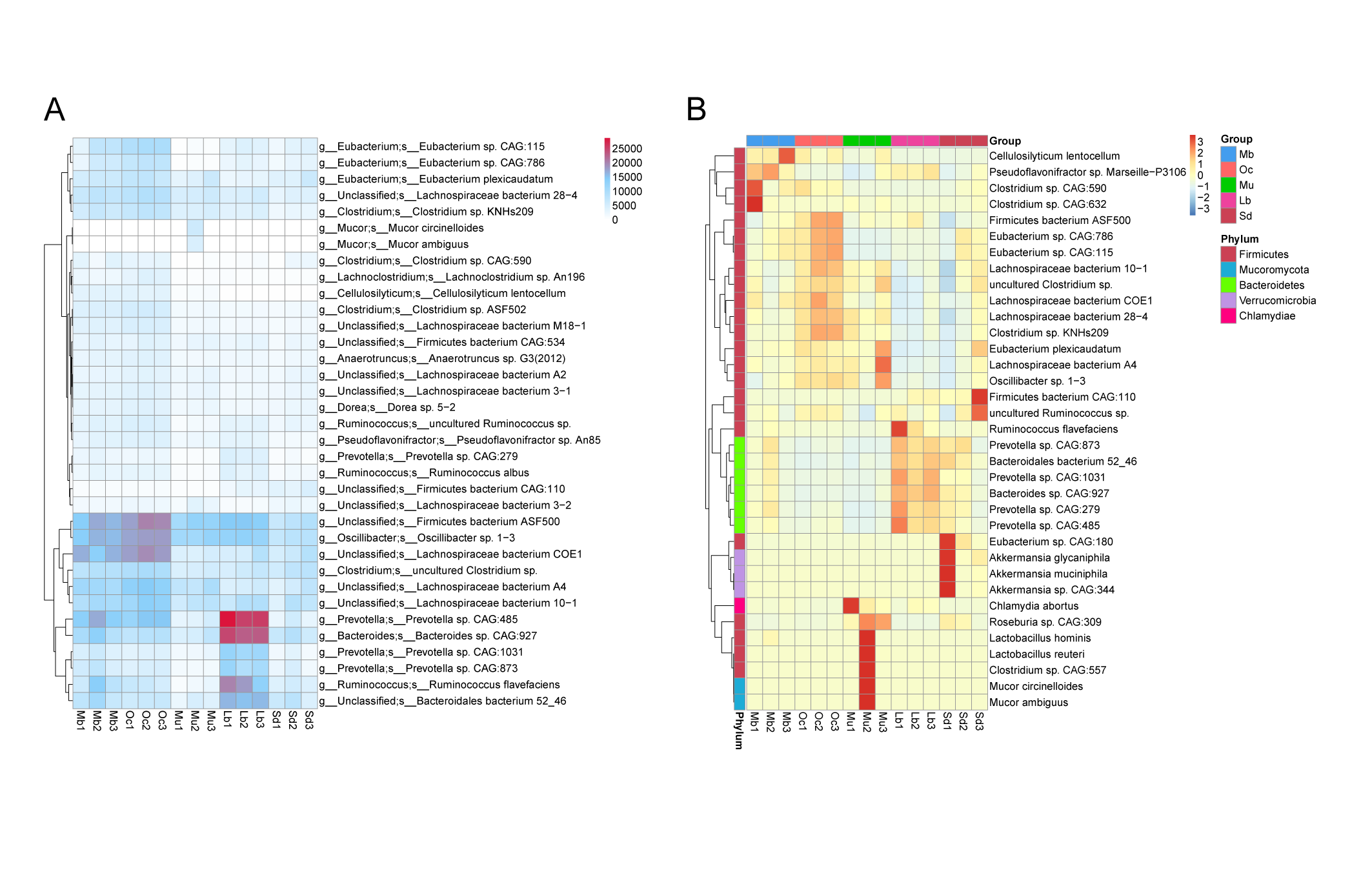

Supplement: Supplementary file 2 [file Data_Sheet_1.ZIP › Figure S2.tif]

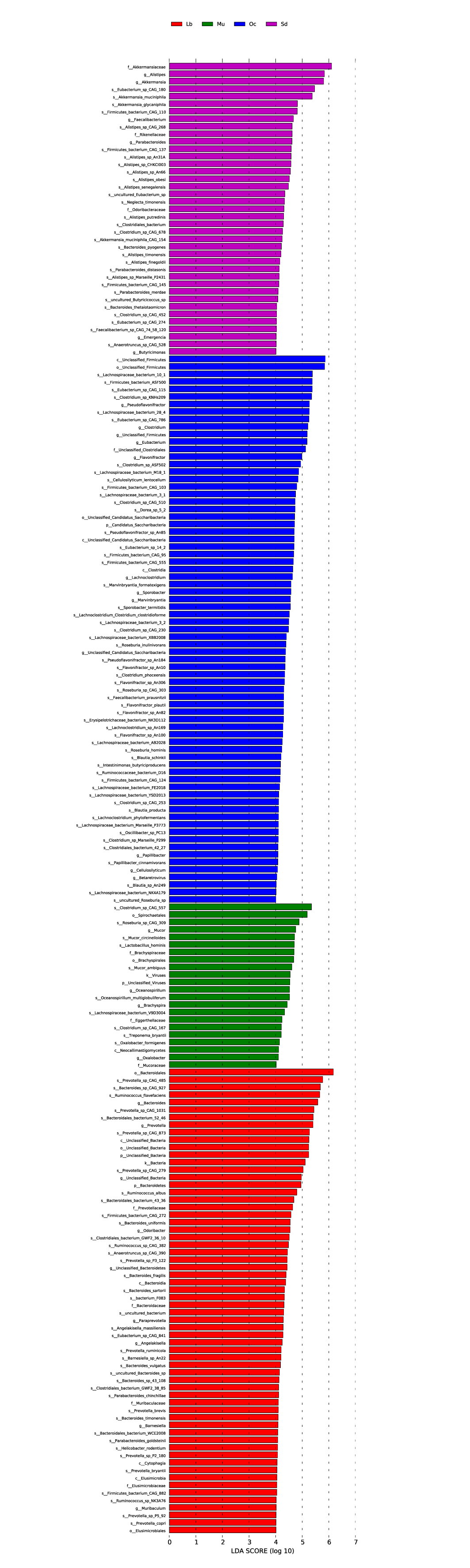

Supplement: Supplementary file 2 [file Data_Sheet_1.ZIP › Figure S3.tif]

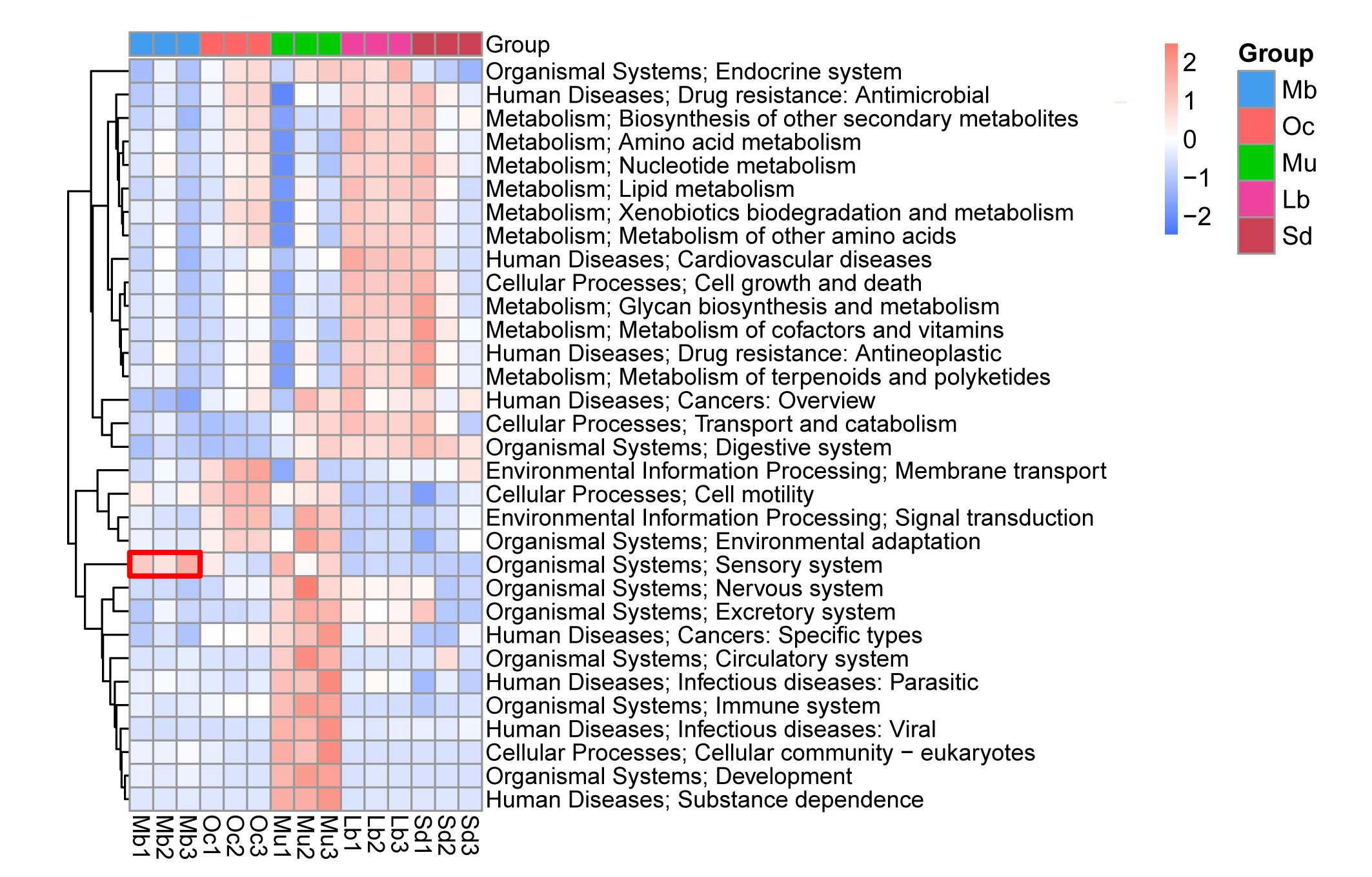

Supplement: Supplementary file 2 [file Data_Sheet_1.ZIP › Figure S4.tif]

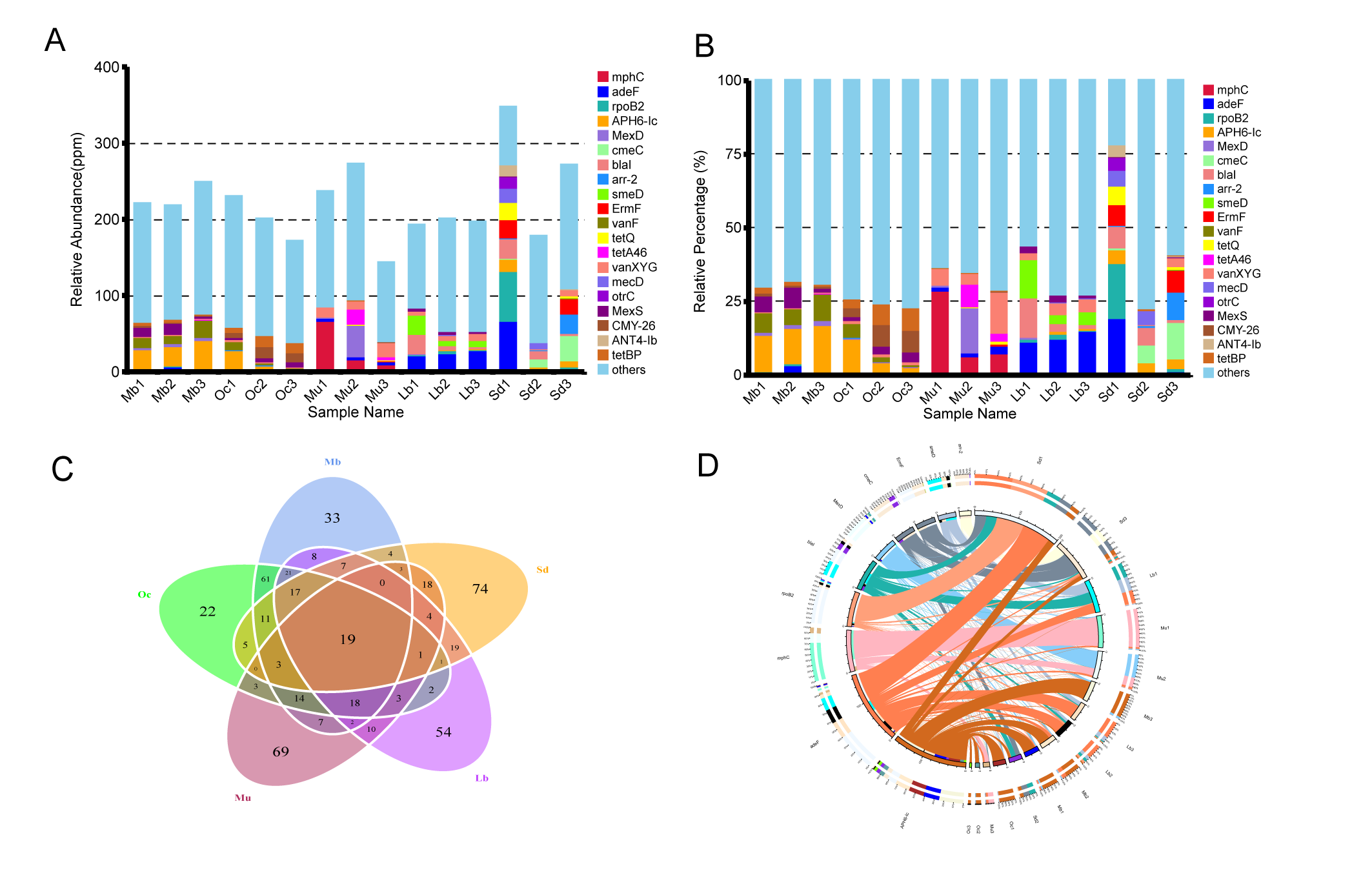

Supplement: Supplementary file 2 [file Data_Sheet_1.ZIP › Figure S5.tif]
